# Drift velocity of bacterial chemotaxis in dynamic chemical environments

**DOI:** 10.1098/rsta.2024.0261

**Published:** 2025-09-11

**Authors:** Jason Singh Bains, Andrew William Baggaley, Ottavio Alfred Croze

**Affiliations:** ^1^ School of Mathematics, Statistics and Physics, Newcastle University, Newcastle upon Tyne, Tyne and Wear NE1 7RU, UK

**Keywords:** microswimmers, bacteria, chemotaxis, active matter, active fluids, microbiology, microbial ecology

## Abstract

Chemotaxis allows swimming bacteria to navigate through chemical landscapes. To date, continuum models of chemotactic populations (e.g. Patlak–Keller–Segel models) have considered bacteria responding only to spatial chemical gradients. In these models, chemotactic advection is modelled through a drift velocity proportional to the spatial chemical gradient. In nature and industry, however, bacterial populations experience dynamic, spatio-temporally varying chemical environments, such as the neighbourhood of lysing phytoplankton cells. Recent analyses have shown how temporal gradients can ‘confuse’ individual bacteria, impacting the precision of their gradient estimation. However, very few studies have considered how temporal gradients influence the chemotactic drift velocity of whole populations. Here, we use Monte Carlo simulations to infer the drift velocity of a population when both spatial and temporal gradients are present. We propose an ansatz for the drift velocity, which fits the simulations well. This ansatz allows us to account for how temporal gradients can significantly impact chemotaxis of bacterial populations up a spatial gradient. We explore the consequences of this new effect through a Patlak–Keller-Segel type model applied to single decaying and oscillating pulses of chemoattractant. Finally, we discuss possible biological consequences of our results and extensions of our modelling framework.

This article is part of the theme issue ‘Biological fluid dynamics: emerging directions’.

## 1. Introduction

Microswimmers are an important constituent of biologically active fluids [1]. Swimming is often coupled with the ability to direct motion in response to environmental stimuli (taxis), which confers biological advantage [2]. A prototypical taxis is chemotaxis, which allows navigation up chemical gradients (positive chemotaxis) to increase exposure to beneficial chemicals, e.g. nutrients. It also allows movement down chemical gradients (negative chemotaxis), reducing exposure to harmful chemicals [3]. Examples of chemotactic microswimmers include bacteria [4], microalgae [5] and sperm [6]. Recently, chemotaxis has also been demonstrated in artificial microswimmers [2].

The chemotaxis of bacteria has been extensively studied, in particular with reference to the model species *Escherichia coli* [7]. This microorganism swims by means of helical flagella distributed across the cell body and driven by molecular motors [4]. When the motors turn counterclockwise, the flagella form a helical bundle and the bacterium swims forward; when one or more motors turn clockwise, the flagellum they are spinning unbundles, and the resulting torque causes the bacterium to tumble [4]. This ‘run and tumble’ locomotion is key to the chemotaxis of *E. coli* and similar bacteria [7]. In the absence of chemical gradients, run and tumble motion results in a random walk [8]. However, in the presence of a gradient, runs are on average extended when a bacterium is travelling up the gradient, resulting in biased random walk that transports bacteria up the gradient [8]. Other bacterial species are also able to perform chemotaxis by similar mechanisms [9].

The molecular basis of this motion and its consequences at the population level have been studied extensively experimentally, both at the single cell level and for populations. Similarly, theoretical studies abound. The most common population-scale models are modifications of the model originally proposed by Patlak [10], and Keller & Segel [11]. A key element of these models is that the bacterial population is chemotactically advected up/down gradients through a drift velocity given by


(1.1) $$v_{d}=\chi \nabla c,$$

where χ is the chemotactic sensitivity parameter and c is the concentration of an attractant or repellent chemical, which we will henceforth denote generally as ‘chemo-effectors’. There are various Patlak–Keller–Segel models with different forms of χ to account for various physical phenomena [12,13], though not all are specific to run and tumble chemotaxis. While Keller and Segel’s original drift velocity was phenomenological, it is possible, in the limit of weak gradients, to derive it analytically. This was first achieved by De Gennes [14], and then extended by Locsei to include the effects of rotational diffusion and persistence of direction [15]. Seyrich *et al.* [16] derived a drift velocity model that incorporates rotational diffusion and directional persistence, accounting for logarithmic sensing and angular bias. This approach accounts for the fact that bacteria sense approximately logarithmic concentration gradients and adjust their mean reorientation angle based on these gradients. Numerous other studies have developed modified drift speed models for run and tumble bacterial chemotaxis in fixed spatial gradients [17–19].

**Figure 1.**
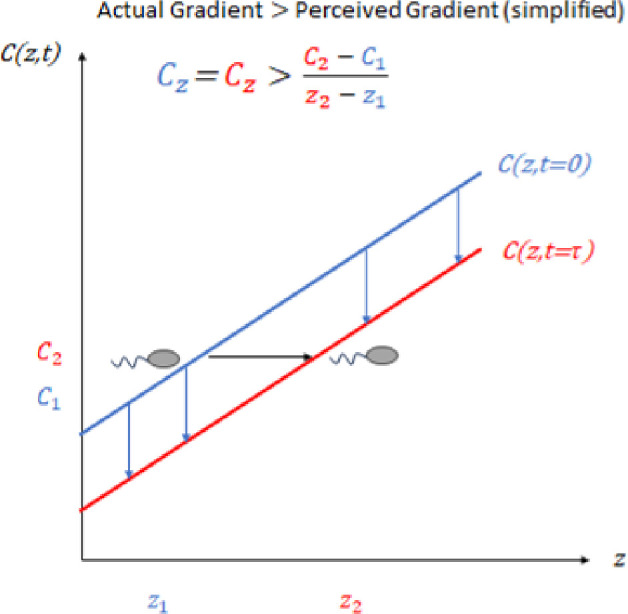
Bacteria swimming in a chemo-effector gradient, which is spatially linear but temporally decreasing in time, perceive a shallower gradient due to the negative temporal variation of concentration.

Bacteria in natural and industrial environments, experience chemical landscapes where both spatial and temporal gradients are present. Indeed, recent work by Hein *et al.* [20] has highlighted how temporal variations in chemicals impact the limits of chemo-sensing in dynamic environments: bacteria can be ‘fooled’ by dynamic gradients as seen in figure 1. This is particularly relevant to bacteria like *E. coli*, which are too small to sense gradients directly [21], and so must make temporal comparisons of chemo-effector levels over time in order to reconstruct the gradient [22]. In view of this, it would be useful to derive an expression for the chemotactic drift velocity of bacterial populations in dynamic chemo-effector environments, i.e. where chemo-effector levels change in both time and space, which can be used in continuum models.

Brumley *et al.* have derived an expression for the drift velocity of *Vibrio* spp. bacteria in spatio-temporally varying environments, which could be (but was not) implemented in Patlak–Keller–Segel models [23]. This was based on a simplified model accounting for gradient measurements over ‘run-reverse’ events, but neglecting the effects of chemotactic memory. To account for memory effects in bacterial populations in temporal gradients we could follow the analytical approach of [14,15]. This involves using a continuum model of chemotactic memory, and its influence on run/tumble ratios, to derive how the sensitivity parameter χ relates to the chemotactic response function R(t). This function models how bacteria respond to chemical stimuli over a characteristic chemical memory time. It can be measured experimentally, for example the response function for *E. coli* in aspartate was measured by Segall *et al.* [24]. The response function determines the chemotactic bias (run/tumble ratio)


(1.2) $$\Delta (t)=\int_{-\infty }^{t}c\left(t^{\prime }\right)R\left(t-t^{\prime }\right)dt^{\prime },$$

which modulates the base tumble rate $\lambda_{0}$, so that the effective tumble rate at time *t* is given by $\lambda (t)=\lambda_{0}e^{-\Delta (t)}$, assuming a Poisson distribution of tumbles. To evaluate the drift velocity, we can determine the expectation value of the bacterial position at the end of a run [15] or, alternatively, we can compute the net run duration [25] or tumble rate [16]. In all cases, the derivation proceeds by Taylor expanding the concentration inside equation (1.2) and then averaging over all bacterial paths. For example, the derivation of Locsei [15], extended to include temporal gradients, leads to


(1.3) $$\begin{array}{lcr} & v_{d}∼\int_{0}^{t}{\left\langle \nabla c\;w(t)z(t^{\prime }-T)+\frac{\partial c}{\partial t}w(t)(t^{\prime }-T)\right\rangle }_{\text{paths}}dt^{\prime }, & \end{array}$$

where angled brackets denote an average over all bacterial swimming paths (a path integral), w(t)=dz/dt is the derivative of bacterial position z along the gradient, and T is a delay time, see [15] for more details. When temporal gradients are absent (∂c/∂t=0), or when these are small compared with the spatial ones, the second term in the sum in equation (1.3) vanishes and it is possible to make analytical progress [14,15]. However, when temporal gradients are significant, these contribute to modifying the bacterial paths, making the calculation of the path integral in equation (1.3) non-trivial. In particular, it is not possible to split the path integral in (1.3) into two, as erroneously assumed by [25].

Given the challenge of evaluating the drift velocity, $v_{d}$, analytically, in this work we set out to simulate the bacterial paths directly by Monte Carlo simulations. We propose an ansatz to $v_{d}$, which fits the simulation results well, and introduces a new temporal chemotactic coefficient to complement the well-known spatial coefficient. Then, inspired by the analysis of [20] for single bacteria, we explore the dependence of the temporal chemotactic coefficient on swimming speed. Finally, we apply the new expression for $v_{d}$ to a modified Patlak–Keller–Segel model. This allows the prediction of bacterial population responses to time-varying pulses. An expression for the chemotactic drift velocity as a function of spatial and temporal gradients, as we obtain in this work, is essential for continuum modelling of chemotactic bacteria. This allows numerical modelling of large populations in various chemo-effector environments at low computational cost compared with agent-based modelling, which simulates many individual bacteria directly. This can have many practical applications, such as modelling plant–bacteria interactions in soil [26].

## 2. Monte Carlo simulations

As an alternative to a challenging analytical approach, we set out to evaluate the drift velocity numerically using Monte Carlo simulations. We followed N=1000 bacteria in an infinite two-dimensional domain (away from boundaries) to establish their paths when placed in a gradient varying spatially and temporally.

### (a) Chemotactic response function

We used the continuum approach to chemosensing, meaning each bacterial path is influenced by the bias given in equation (1.2). This required choosing a chemotactic response function to use in our simulations. Following [15], we used:


(2.1) $$\begin{array}{r} R(t)=\left\{ \begin{array}{ll} \frac{R_{0}}{\tau }\sin(\frac{\pi t}{2\tau }) & 0\leq t\leq 4\tau \\ 0 & 4\tau <t, \end{array}\right. \end{array}$$

where $R_{0}$ is a constant quantifying the strength of response and $\tau =1/\lambda_{0}$ is the bacterial run time. We chose this form as it is computationally convenient, while satisfying the experimentally observed features of bacterial chemotactic response functions [24], recently summarized by Seyrich *et al.* [16]. These features are as follows: the function R(t) is non-zero over a characteristic time $t_{m}$, which represents the time over which a cell has memory of its chemical environment. For the case of *E. coli*, considered here, $t_{m}=4\tau s$ [24]. Secondly, R(t) must reflect perfect adaptation: the tumble rate is only modulated by the differences in sensed chemo-effector levels over the memory time. There is no direct dependence on absolute chemo-effector levels. Mathematically, this implies $\int_{0}^{\infty }R(t)dt=0$, which we can see the form of equation (2.1) satisfies by definition. Finally, the response function must be inversely proportional to some adaptation concentration $c_{a}$ [24], meaning we could rewrite our response function as


(2.2) $$\begin{array}{lcr} & R(t)=\frac{\tilde{R}(t)}{c_{a}}. & \end{array}$$

We could thus write the chemotactic bias in equation (1.2) as


(2.3) $$\Delta (t)=\frac{1}{c_{a}}\int_{-\infty }^{t}c\left(z(t^{\prime }),t^{\prime }\right)\tilde{R}\left(t-t^{\prime }\right)dt^{\prime },$$

where, for the purpose of this paper, we considered a concentration c that varies with time t as well as position along the gradient z. The integral given in equation (2.3), whose numerical evaluation is required for the simulations, can be simplified analytically. Consider the substitution $u=t-t^{\prime }$ in (2.3). A bacterium is small on the scale of the gradient, so we can Taylor expand the concentration c as


(2.4) $$\begin{array}{lcr} & c(z(t-u),t-u)\approx c(z(t),t)+\frac{\partial c(z(t),t)}{\partial z}\left(z(t-u)-z(t)\right)-\frac{\partial c(z(t),t)}{\partial t}u, & \end{array}$$

disregarding second and higher order terms. We then substituted equation (2.4) into (2.3), using the perfect adaptation condition $\int_{0}^{\infty }\tilde{R}(u)du=0$, to arrive at the new expression for the chemotactic bias


(2.5) $$\Delta (t)=\frac{1}{c_{a}}\left(\frac{\partial c}{\partial z}\int_{0}^{\infty }\tilde{R}\left(u\right)z\left(t-u\right)du-\frac{\partial c}{\partial t}\int_{0}^{\infty }\tilde{R}\left(u\right)udu\right).$$

Thus, because the second integral analytically evaluates to a constant, only $\int_{0}^{\infty }\tilde{R}\left(u\right)z\left(t-u\right)du$ needs to be estimated numerically. From this integral, we can also estimate the maximum possible bias, which occurs when cells swim directly up the gradient. We substitute z=vt into (2.5) to obtain


(2.6) $${\left|\Delta (t)\right|}_{max}=\frac{1}{c_{a}}\left(v_{s}\left|\frac{\partial c}{\partial z}\right|+\left|\frac{\partial c}{\partial t}\right|\right)\left|\int_{0}^{\infty }\tilde{R}\left(u\right)udu\right|.$$

### (b) Simulation details

Now that we have an expression for the bias, we can calculate the non-dimensional tumble rate, $\lambda^{*}(t)=\lambda (t)/\lambda_{0}$, and probability ℙ(tumble) that bacteria will tumble in a given time interval dt:


(2.7)λ∗(t)=e−Δ(t)(2.8)P(tumble)=1−e−λ∗(t).

Numerically we generated a pseudorandom number using the MATLAB random number generator for each bacterium, denoted X, where X∼U(0,1); each cell for which X≤ℙ(tumble) will tumble. Our model also includes directional persistence and rotational diffusion in two dimensions. On tumbling, bacteria pick an angle $\psi_{T}$ to the current run direction, such that $\langle \cos(\psi_{T})\rangle =\alpha_{p}$, where $\alpha_{p}$ is the persistence parameter. In between tumbles, cells will also be reoriented by rotational diffusion, with diffusivity $D_{r}$. We used $D_{r}=0.062{\text{ radians}}^{2}{\text{s}}^{-1}$ for all simulations, which is the value Berg estimated for *E. coli* cells swimming in room temperature water [27]. Therefore each tumbling bacterium has their orientation angle updated according to


(2.9) $$\begin{array}{rl} \phi_{i}(t+dt) & =\phi_{i}(t)+\Delta \phi , \end{array}$$

where, when tumble events occur, $\Delta \phi ∼(-1{)}^{j}N(\cos^{-1}(\alpha_{p}),\sigma^{2})$, where N is a normal distribution for the persistence cosine, for simulation purposes, $\alpha_{p}$ and $\sigma^{2}$ were taken from measurements of *E. coli* in [8]. Each tumbling bacterium has its Δϕ generated randomly from this distribution. The index j∼U{0,1} establishes if reorientation in two dimensions occurs clockwise (j=1) or anticlockwise (j=0) relative to the previous orientation. When bacteria are not tumbling, their orientations are updated to account for rotational diffusion, so that $\Delta \phi ∼N\left(0,2D_{r}dt\right)$, where N is a normal distribution with zero mean and standard deviation $2D_{r}dt$. Correspondingly to these reorientations, at the beginning of the following time step, bacterial positions are updated according to


(2.10) $$x_{i}(t+dt)=x_{i}(t)+v_{s}dtp_{i}(t),$$

where $p_{i}(t)=\left(\cos\;\phi_{i},\sin\;\phi_{i}\right)$.

### (c) Choice of gradient, non-dimensionalization and parameters

To test our approach, we simulated bacteria swimming in a concentration profile that is linear in space and time:


(2.11) $$c(z,t)=c_{0}+az+bt,$$

where $c_{0}$ is a reference concentration, and ∂c/∂z=a and ∂c/∂t=b are spatial and temporal gradient strengths, respectively. We note that, while we consider two-dimensional bacterial dynamics, this concentration profile is one-dimensional and grows spatially in the z-direction. Henceforth, we non-dimensionalized our model as follows: $c^{*}=c/c_{0}$, $t^{*}=t/\tau$, $z^{*}=z/\tau v_{0}$ and $V=v_{s}/v_{0}$. Here τ is the average run duration, $\tau =1/\lambda_{0}$, and $v_{0}$ is the mean swimming speed of the bacteria (the base swimming speed for chemokinetic bacteria [25]). Assuming the average run duration is of the order 1s, then it will also be reasonable to assume the dimensionless rotational diffusivity parameter to be $D_{r}^{*}=0.062{\text{ radians}}^{2}$. This, dropping asterisks for notational clarity, leads to the following non-dimensional chemical profile


(2.12) c(z,t)=1+αz+βt,

where the non-dimensional spatial and temporal gradient strengths are $\alpha =a\tau v_{0}/c_{0}$ and $\beta =b\tau /c_{0}$, respectively. We will consider a chemical profile that resets instantaneously at the beginning of each new run, that is, the profile perceived by a cell with position $z_{c}$ and time $t_{c}$ at the start of a run, is given by


(2.13) $$\begin{array}{lcr} & c(z,t)=1+\alpha (z-z_{c})+\beta (t-t_{c}). & \end{array}$$

This resetting is possible because the concentration profile is linear, so cells in successive runs, i.e. after each tumble, experience the same chemical environment. It allows us to avoid having to explicitly account for an adaptation concentration $c_{a}$, which would grow to a point at which chemotaxis was not viable. Further, in the simulation, chemotactic memory from the previous $\tau_{m}$ seconds is stored and fed into the next run, so that chemotactic adaptation is preserved.

As the chemical profile in our simulation ‘resets’ at the start of each run for each cell, we could see from equation (2.13) that c(t)≈1 for the majority of the simulation, so in equation (2.2) and following it is reasonable to set $c_{a}=c_{0}$.

All bacteria were located initially at (0,0) and their chemotactic memories are ‘blank’. Each bacterium i was initialized with orientation angle $\phi_{i}∼U[0,2\pi ]$ so that its orientation vector was $p_{i}=\left[\cos(\phi_{i}),\sin(\phi_{i})\right]$. Time was discretized into even blocks of dt=0.1. At the end of each block of time the chemotactic bias for each individual bacterium was given by the non-dimensional version of equation (2.5),


(2.14) $$\Delta (t)=\alpha \int_{0}^{\infty }\tilde{R}\left(u\right)z\left(t-u\right)du+\frac{8\tilde{R_{0}}}{\pi }\beta ,$$

where we had evaluated the second integral in equation (2.5) using the response function (2.1). The maximum bias is then given by (non-dimensionalizing equation (2.6))


(2.15) $$\Delta (t{)}_{max}=\frac{8\tilde{R_{0}}}{c\pi }\left(v_{s}\alpha -\beta \right).$$

Once a simulation was complete, the drift velocity was evaluated from


(2.16) $$v_{d}=\frac{\left\langle \Delta x\right\rangle }{\tau_{max}},$$

where Δx is the bacterial displacement, $\tau_{max}$ is the simulation time and angled brackets denote an average over all bacteria.

## 3. Results and ansatz for the drift velocity

**Figure 2.**
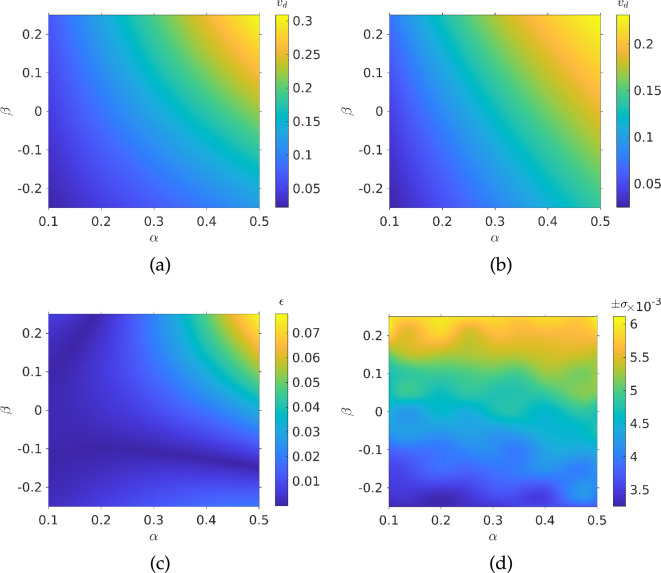
Drift velocity of a population of bacteria from Monte Carlo simulations: (*a*) interpolated simulation results; (*b*) ansatz from equation (3.1) plotted for the same values of α and β; (*c*) 2-norm of error between simulation and ansatz; (*d*) interpolated standard deviations from each simulation run. Parameters used: N= 1000, $\tau_{max}=10^{4}$, V=1, $\alpha_{p}=0.5$, σ=±13π/90 radians, $D_{r}=0.062{\text{ radians}}^{2}$.

The Monte Carlo simulations were initially run over a range of small values of α and β. Results are presented in figure 2*a*. We see that the drift speed depends on both spatial and temporal gradients. The largest value is when both α and β are large. The smallest value is when α is small and β has the largest negative value. The simulations can predict the dependence of the drift speed on the gradients. However, it would be time consuming to have to run a simulation afresh to obtain a drift velocity for every set of chemotactic parameters. Instead, we propose the following ansatz to describe the drift velocity:


(3.1) $$\begin{array}{lcr} & v_{d}=\left(1+\tilde{\chi_{1}}\beta \right)\tilde{\chi_{0}}\alpha . & \end{array}$$

**Figure 3.**
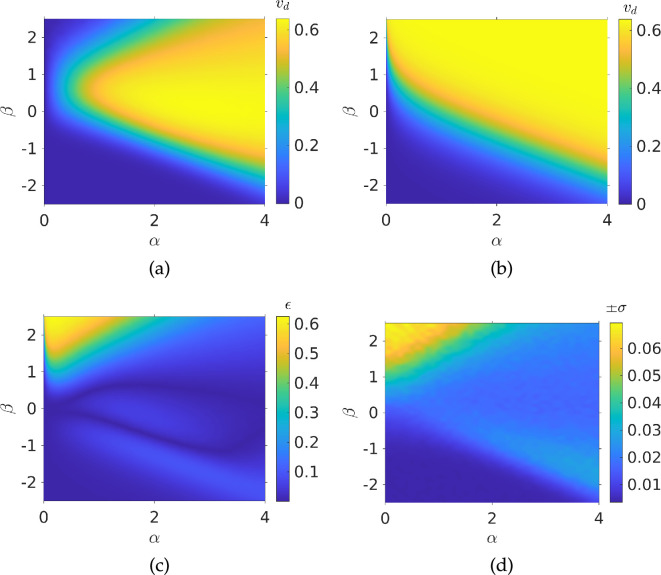
Drift velocity of a population of bacteria from Monte Carlo simulations: (*a*) interpolated simulation results; (*b*) ansatz from equation (3.5) plotted for the same values of α and β; (*c*) 2-norm of error between simulation and ansatz; (*d*) interpolated standard deviations from each simulation run. Parameters used: N= 1000, $\tau_{max}=10^{4}$, V=1, $\alpha_{p}=0.5$, σ=±13π/90 radians, $D_{r}=0.062{\text{ radians}}^{2}$.

This works well for small gradients, but, to describe a broad range of environmental gradients that bacteria may be subject to, it is of interest to run simulations for larger values of α and β, see figure 3*a*. Comparing the latter with figure 2*b*, we see that our ansatz begins to break down for large gradient values, and it must thus be modified to be applicable in this regime.

From figure 3*a*, we notice that sufficiently strong spatial and temporal gradients lead to a much larger growth in drift velocity followed by saturation at some maximum speed, which we denote as $v_{d\infty }$. Long *et al.* [28] predicted this speed in three dimensions would approach V/2, where V is the dimensionless speed of the bacterium. In two dimensions, we expect $v_{d\infty }<3V/4$. In what follows, we will write $v_{d\infty }=v_{\infty }V$ where the parameter $v_{\infty }\leq 1$. We obtained $v_{d\infty }=0.63$ from a fit to the data in figure 3*a* (deriving this bound analytically, with an account of directional persistence and rotational diffusion, is beyond the scope of this work). Beyond this maximum speed, sufficiently strong spatial or temporal gradients cause the drift speed to decay to zero. We will first consider the limit of purely spatial gradients. In this case, we can define the base chemotactic drift velocity to be


(3.2) $$v_{d}^{s}=v_{\infty }V\tanh\left(\frac{\tilde{\chi_{0}}}{v_{\infty }V}|\alpha |\right)\frac{\alpha }{|\alpha |},$$

where we model the change in the drift speed as a hyperbolic tangent.

Moving on to consider temporal gradients, we can think of equation (3.1) as a Maclaurin expansion of the exponential function, so that the drift velocity can be written as $v_{d}=v_{d}^{s}\exp\;\left(\tilde{\chi_{1}}\beta \right)$, where $v_{d}^{s}$ is given by equation (3.2). This expression for $v_{d}$, however, does not describe the simulations in figure 3*a*, as it grows unboundedly with temporal gradients, instead of reaching a finite value, with maximum $v_{\infty }V$. To amend this, we include a logistic function in the final ansatz, which then becomes


(3.3)vd=ϵ(α,β,c)v∞V[1+(1vd0−1)e−χ1~β]−1α|α|,(3.4)vd0=tanh⁡(χ0~v∞V|α|),

where ${\tilde{\chi }}_{0}$ and ${\tilde{\chi }}_{1}$ are dimensionless chemotactic parameters, whose meaning is discussed below, and ϵ is a function that captures the sensing thresholds for bacteria that will be discussed in the next section. It can be easily checked that equation (3.3) reduces to the purely spatial drift in equation (3.2) (multiplied by ϵ) when β≪1. The new ansatz also has the desired property of saturating to $\epsilon v_{\infty }V\alpha /|\alpha |$ when β≫1.

Comparing the simulation results of figure 3*a* and the drift velocity predicted by the ansatz, shown in figure 3*b*, we see that there is broad qualitative agreement between simulations and ansatz predictions. Figure 3*c*, which displays the 2-norm of the error between the simulation and the ansatz prediction, shows that this agreement is quantitative for a large region of parameter space. However, this breaks down where α≪|β|; we will see in the next section how this is due to the limits of bacterial sensing, quantified by the threshold function ϵ. Finally, we note that the standard deviation of the drift velocity from our simulations was at most $7\times 10^{-3}$, even as the drift speed decays to 0, see figure 3*d*. We can thus be sure that any features in our results are not due to fluctuations in the simulations. To motivate our ansatz, it is helpful to re-dimensionalize equation (3.3) and consider general spatial and temporal gradients, so that


(3.5)vd=ϵ(‖∇c‖,∂c/∂t,c)v∞vs[1+(1vd0−1)exp⁡(−χ1∂c∂t)]−1∇c‖∇c‖,(3.6)vd0=tanh⁡(χ0v∞vs‖∇c‖),

where $\chi_{0}$ and $\chi_{1}$ are chemotactic parameters. In the case of no temporal gradients and ‖∇c‖≪1, equation (3.5) becomes $v_{d}=\chi_{0}\nabla c$, which is the standard Patlak–Keller–Segel model. In analytical derivations of the chemotactic drift velocity it has been shown that


(3.7) $$\chi_{0}=\frac{v_{s}^{2}\tau }{c_{a}}\int_{0}^{\infty }\tilde{R}(u)f(u)du,$$

where the function f(u), including the effects of directional persistence and rotational diffusion, was determined by Locsei [15]. The temporal chemotactic parameter $\chi_{1}$ has not been obtained previously, analytically or otherwise. It might be expected that this parameter should, like $\chi_{0}$, depend on the chemotactic response function $\tilde{R}(t)$. Indeed, we found that the following form fits our simulation data well:


(3.8) $$\begin{array}{lcr} & \chi_{1}=\frac{\tau }{c_{a}}\left|\int_{0}^{\infty }\tilde{R}(u)udu\right|. & \end{array}$$

The expression $\left|\int_{0}^{\infty }\tilde{R}(u)udu\right|$ is the same as appears in equation (2.6) for the maximum bias. This makes sense, as negative/positive temporal variations uniformly bias runs to be shorter/longer, and we see this is reflected in the temporal contribution to the drift velocity. Next, we define two characteristic times $\delta_{0}=\tau \int_{0}^{\infty }\tilde{R}(u)f(u)du$ and $\delta_{1}=\left|\int_{0}^{\infty }\tilde{R}(u)udu\right|$ and make the assumption that $c_{a}=c(t)$, which is reasonable, as adaptation occurs over the run time [16]. We can then write our two chemotactic parameters as $\chi_{0}=v_{s}^{2}\delta_{0}/c$ and $\chi_{1}=\delta_{1}/c$. Substituting these expressions into equation (3.5) the ansatz for the drift velocity becomes


(3.9)vd=ϵ(‖∇c‖,∂c/∂t,c)v∞vs[1+(1vd0−1)exp⁡(−δ1c∂c∂t)]−1∇c‖∇c‖,(3.10)vd0=tanh⁡(vsδ0v∞‖∇c‖c).

### (a) Sensing thresholds and ansatz correction

We need to address the breakdown of the drift velocity predicted by the ansatz when ‖∇c‖≪|∂c/∂t|, which can be observed in figure 3*c*. In simulations (figure 3*a*), positive temporal gradients |∂c/∂t| approach some value κ‖∇c‖, where κ is a critical constant. After this, the drift velocity smoothly transitions to zero. However, as discussed above our ansatz saturates to a maximum speed, as shown in figure 3*b*. The drop to zero in simulations, not captured by our ansatz, is due to the fact that, when |∂c/∂t|≫‖∇c‖, the chemotactic bias will be nearly entirely determined by |∂c/∂t|, as can be seen in equation (2.5). Thus, swimming up or down the spatial gradient provides only minor modulations to the tumble rate, resulting in a negligible chemotactic drift speed. This effect is offset by faster swimming, as first proposed by Hein *et al.* [20], who predicted that faster swimming bacteria should be able to better detect the direction of a spatial gradient, before temporal changes confound this. The gradient sensed by a single bacterium over the course of a sensing interval Δt, is given by $\Delta t\left(v_{z}\|\nabla c\|+\partial c/\partial t\right)$ where $v_{z}$ is the swimming speed in the gradient direction. If this speed averaged over the whole population is denoted by $\left\langle v_{z}\right\rangle$, we can say that, when $\|\nabla c\|\ll |\partial c/\partial t|/\left\langle v_{z}\right\rangle$, chemotaxis is not possible. Since $\left\langle v_{z}\right\rangle$ depends on $v_{s}$ we can rewrite our critical value κ as $\kappa_{c}/v_{s}$. On this basis, the sensing threshold function is assumed to take the form


(3.11) $$\begin{array}{lcr} & \epsilon \left(\|\nabla c\|,\partial c/\partial t,c\right)=\left[1-\tanh\left(\frac{\kappa_{c}|\partial c/\partial t|}{v_{s}\|\nabla c\|}\right)\right]. & \end{array}$$

**Figure 4.**
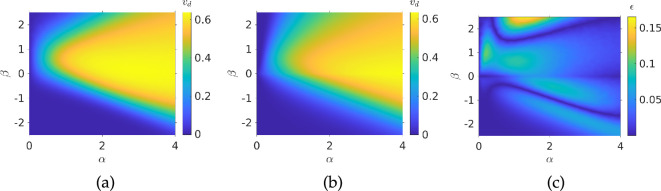
Fitting the ansatz from equation (3.9) to simulation data from figure 3 with sensing threshold given by equation (3.11) and g(c)=1: (*a*) simulation data; (*b*) ansatz plotted with fitted value $\kappa_{c}=0.3014$; (*c*) 2-norm of error between simulation and ansatz. Parameters used: N= 1000, $\tau_{max}=10^{4}$, V=1, $\alpha_{p}=0.5$, σ=±13π/90 radians, $D_{r}=0.062{\text{ radians}}^{2}$.

We can validate this assumption by fitting our ansatz to the drift velocity in order to determine $\kappa_{c}$ and see if that results in better agreement for our ansatz prediction with the simulation data. The fit provides $\kappa_{c}=0.3014$*.* The ansatz prediction and resulting error are shown in figure 4. If we compare figures 3*c* and 4*c*, we see that this threshold function leads to much better agreement, with maximum error of 0.16 compared with 0.6 and generally lower errors across different values of α and β.

In addition to the effect of speed, we also need to account for the fact that bacteria need a certain minimum concentration, $c_{l}$, of chemoattractant to be able to chemo-sense; below this threshold there is not enough chemo-effector to make meaningful comparisons of concentration [29]. There is also a maximum concentration $c_{h}$ above which all receptors are saturated, inhibiting chemotactic response [29]. Therefore, for $c<c_{l}$ and $c>c_{h}$, the chemotactic drift velocity will be zero. There are many methods to implement these sensing thresholds, but we take inspiration from Seyrich *et al.* [16] and choose hyperbolic tangent functions (they used such thresholds on the tumble rate not the drift velocity, but similar considerations apply). Implementing these thresholds our sensing threshold function becomes


(3.12) $$\epsilon \left(\|\nabla c\|,\partial c/\partial t,c\right)=\left[1-\tanh\left(\frac{\kappa_{c}|\partial c/\partial t|}{v_{s}\|\nabla c\|}\right)\right]\left[1-\tanh\left(\frac{c}{c_{h}}\right)\right]\tanh\left(\frac{c}{c_{l}}\right),$$

where we note that the thresholds also prevent the drift velocity from becoming singular as c→0.

### (b) Speed dependence of temporal effects on drift velocity

**Figure 5.**
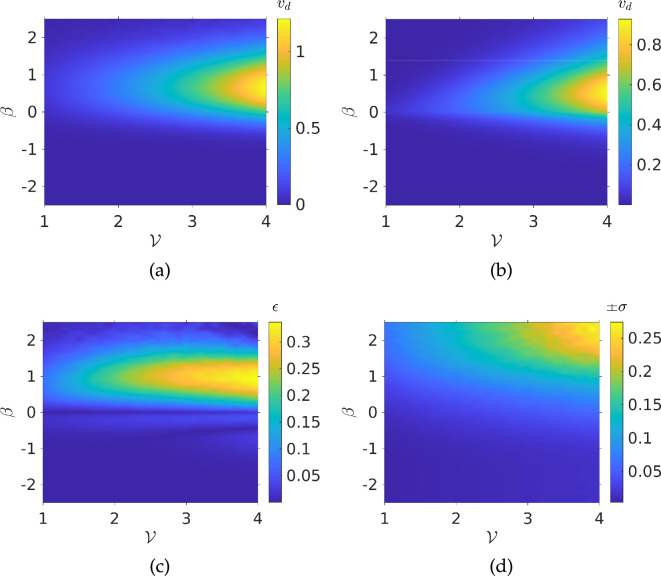
Speed dependence of the drift velocity: (*a*) interpolated simulation run result; (*b*) ansatz from equation (3.9) plotted for the same values of β and V; (*c*) 2-norm of error between simulation and ansatz; (*d*) interpolated standard deviations from each simulation run. Parameters used: N= 1000, $\tau_{max}=10^{4}$, α=0.1, $\alpha_{p}=0.5$, σ=±13π/90 radians, $D_{r}=0.062{\text{ radians}}^{2}$.

Our newly derived ansatz includes several dependencies on swimming speed. Thus, to be confident in the predictive power of our ansatz, we will test it against simulations when swimming speed is varied. To this end, we ran further Monte Carlo simulations varying the temporal gradient parameter β and the swimming speed V, with non-dimensional spatial gradient held fixed at α=0.1. We then compared the results against the non-dimensional version of equation (3.9), with upper/lower concentration sensing thresholds neglected. The simulation results shown in figure 5 can be seen to agree well qualitatively with equation (3.9) (figure 5*b*), when sensing thresholds are implemented with the same value of $\kappa_{c}$ obtained from the fitting in figure 4. The agreement breaks down quantitatively, as shown in figure 5*c*. This is probably due to imprecision in the determination $v_{\infty }$, on which the ansatz depends and which was fitting to data from figure 3. A more rigorous determination of $v_{\infty }$ would likely remedy this issue, but this is beyond the scope of the work, where we are more interested in the qualitative nature of the drift velocity. Finally, we note that standard deviations are higher in figure 5*d* compared with figure 3*d*, most likely due to the fact that when chemotaxis breaks down, bacteria move purely diffusively, and faster bacteria can spread out faster. However, it is clear from figure 5*d* that this is only an issue at very high speeds and temporal gradients, where the drift velocity is small, so we can still be confident in our results.

## 4. Continuum model

To illustrate the effect of temporal variations on chemotactic populations of bacteria, and to highlight the practical application of the ansatz, we simulated some scenarios, which could be realized in microfluidic experiments. We adopted a continuum approach, in which the transport equations for the chemo-effector concentration c and bacterial density b are given by


(4.1)∂c∂t=−∇⋅Jc+f(c,b)(4.2)∂b∂t=−∇⋅Jb+g(c,b),

where $J_{c}$ and $J_{b}$ are the flux of chemo-effector and bacteria, respectively. The function f(c,b) determines the consumption and production of chemo-effector by bacteria, while g(c,b) determines the growth and death of bacteria. In what follows, we neglected both consumption and growth (f(c,b)=g(c,b)=0), which experimentally would correspond to considering non-metabolizable chemoattractants [30] and experimental timescales short compared with bacterial doubling times. We assumed that the chemo-effector undergoes isotropic diffusion with constant diffusivity $D_{c}$, so that $J_{c}=-D_{c}\nabla c$. The bacterial flux combines a drift due to chemotaxis with diffusion caused by run-and-tumble swimming, so that $J_{b}=v_{d}b-D_{b}\nabla b$, where $D_{b}=v_{s}^{2}\tau /d$ [14], with d being the dimensions of the space, here d=2. To summarize, our transport model becomes


(4.3)∂c∂t=Dc∇2c(4.4)∂b∂t=vs2τd∇2b−∇⋅(vdb)(4.5)vd=ϵ(‖∇c‖,∂c/∂t,c)v∞vs(1+(1vd0−1)exp⁡(−δ1c∂c∂t))−1∇c‖∇c‖,(4.6)vd0=tanh⁡(vsδ0v∞‖∇c‖c),(4.7)ϵ=[1−tanh⁡(κc|∂c/∂t|vs‖∇c‖)][1−tanh⁡(cch)]tanh⁡(ccl).

We considered bacterial dynamics in two-dimensional, with Cartesian coordinates (x,z). The model was solved numerically (see next section for details) on a two-dimensional rectangular domain Ω=[0,L]×[0,L], with no-flux boundary conditions for both chemo-effector and bacterial concentration.

### (a) Non-dimensionalization

Our system of transport equations (4.3)–(4.7) can be non-dimensionalized using the following scalings,


$$\begin{array}{c} \begin{array}{c} \left(x^{*},z^{*}\right)=\frac{1}{\delta_{0}v_{0}}(x,z),\;t^{*}=\frac{t}{\delta_{0}},\;b^{*}=\frac{b}{b_{0}},\\ \left(c^{*},c_{l}^{*},c_{h}^{*}\right)=\frac{1}{c_{0}}(c,c_{l},c_{h}), \end{array} \end{array}$$

where $b_{0}$ and $c_{0}$ are some reference concentrations of bacteria, noting that these are not fixed values and $c_{0}$ need not have the same value as that given in equation (2.11). The speed $v_{0}$ is a reference swimming speed. Times have been rescaled by $\delta_{0}$ and lengths by $v_{0}\delta_{0}$, so that the boundaries extend to $L^{*}=L/\delta_{0}v_{0}$. Thus, our non-dimensional equations of transport are, omitting asterisks for notational clarity,


(4.8)∂c∂t=γ∇2c(4.9)∂b∂t=V2ζ∇2b−∇⋅(bvd)(4.10)vd=ϵv∞V(1+(1vd0−1)exp⁡(−ηc∂c∂t))−1∇c‖∇c‖,(4.11)vd0=tanh⁡(Vv∞‖∇c‖c).(4.12)ϵ=[1−tanh⁡(κc|∂c/∂t|V‖∇c‖)][1−tanh⁡(cch)]tanh⁡(ccl),

with non-dimensional parameters,


$$\begin{array}{c} \begin{array}{c} \gamma =\frac{D_{c}}{\delta_{0}v_{0}^{2}},\;\zeta =\frac{\tau }{d\delta_{0}},\;\eta =\frac{\delta_{1}}{\delta_{0}},\;V=\frac{v_{s}}{v_{0}}, \end{array} \end{array}$$

and non-dimensional no-flux boundary conditions.

## 5. Simulations of dynamic chemical environments

To simulate examples of dynamic chemical profiles, we solved the model equations numerically using a spectral element method [31], with Lagrange basis functions, to discretize our domain at 64 Gauss points per element, which are 10×10 in size. We then advanced in time using the Dopri-54, adaptive, explicit Runge–Kutta scheme [32].

### (a) Chemo-effector pulse

**Figure 6.**
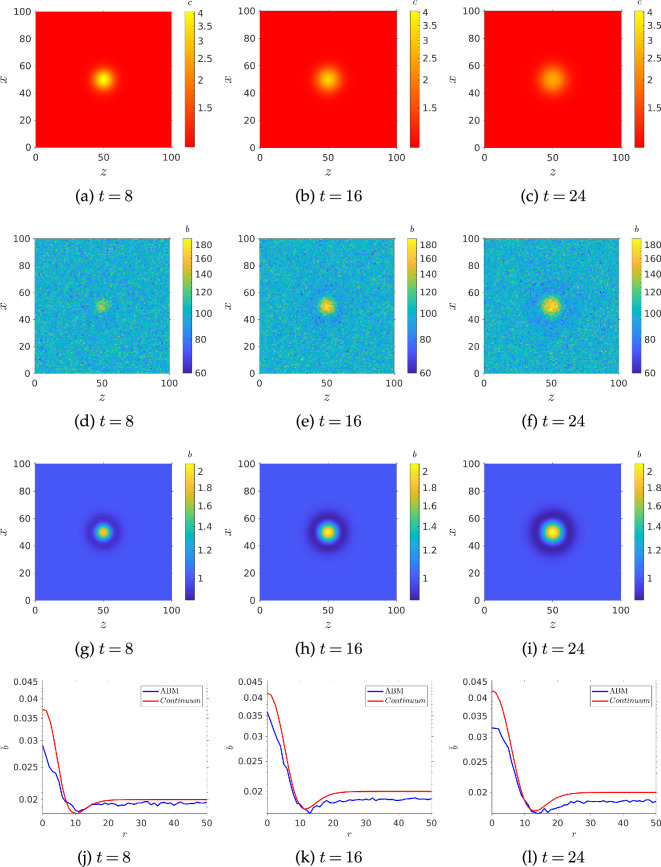
Continuum model versus agent-based model for an initially uniform bacterial population responding to a decaying pulse of chemo effector: (*a*)–(*c*) chemo-effector concentration; (*d*)–(*f*) cell density from agent-based model of bacterial population; (*g*)–(*j*) cell density from continuum model of bacterial population; (*j*)–(*l*) normalized and azimuthally averaged cell concentrations for agent-based model (bars) and continuum model (line). Parameters used: $N=10^{6}$, $\tau_{max}=24$,$\alpha_{p}=0.5$, σ=±13π/90 radians, $D_{r}=0.062{\text{ radians}}^{2}$, $\tilde{R_{0}}=2$, $\zeta =0.5\;,\kappa_{c}=0.3104\;,V=1,\;\eta =5.7875,\;\gamma =0.5,\;k=5,\;\sigma_{c}=2.5,\;v_{\infty }=0.63,\;L=100$.

We shall first consider the case of an initially uniformly distributed bacterial population responding to a single pulse of chemo-effector. In dynamic environments where nutrients are scarce, bacteria often have to navigate through transient chemical landscapes, such as nutrient pulses from lysing algal cells [33]. These pulses diffuse away rapidly creating significant temporal gradients. We will simulate these pulses by choosing our initial conditions to be


(5.1) $$\begin{array}{lcr} & c(x,z,0)=1+k\;\exp\left(-\frac{[(x-L/2{)}^{2}+(z-L/2{)}^{2}{]}^{1/2}}{\sigma_{c}}\right),\;b(x,z,0)=1, & \end{array}$$

where k represents the strength of pulse and σ is its initial spread. Before we use the continuum model to study the pulse, it is important to validate it against an agent-based model (ABM). To that end we will simulate N bacteria, as in §2. We assume perfect specular reflection at boundaries, and approximate the integral in equation (1.2) by interpolating the chemo-effector concentration at bacteria points every dt=0.1 and storing it over the memory time. For computational ease we assume that $\lambda_{0}=1/\delta_{0}$ and use the response function given by


(5.2) $$\begin{array}{r} R(t,c)=\left\{ \begin{array}{ll} \frac{{\tilde{R}}_{0}}{\delta_{0}c}\sin(\frac{\pi t}{2\delta_{0}}) & 0\leq t\leq 4\delta_{0}\\ 0 & 4\delta_{0}<t, \end{array}\right. \end{array}$$

where we made the assumption, as in the continuum case, that $c_{a}=c$. The results of these simulations can be seen in figure 6*d–f*. Bacteria in the ABM were divided into 1×1 bins to calculate cell density. This was compared with the cell density from our continuum model, as can be seen in figure 6*g*–*i*. As we can see from figure 6*j–l*, there is good qualitative agreement between the azimuthally averaged cell density distributions predicted by the agent-based and continuum models. Quantitatively, agreement is worse agreement near the origin, likely due to the fact that the azimuthal averaging area near the origin is very small, so it is hard to get a high level of resolution without simulating a larger number of agents. The disagreement could also be due to imperfect parametrization. As we have already seen, the $v_{\infty }$ parameter would benefit from more careful determination. Also, the ABM population is lower than the continuum model predicts. This is due to the our simplified ABM boundary conditions leading to greater accumulation in the corners of our domain. However, given the qualitative agreement between ABM and continuum model, we can be confident in the results from the latter and use it to study the response of a bacterial population responding to various pulses with reduced computational cost.

Temporal gradients influence bacterial exposure to chemo-effectors, which can be beneficial or deleterious. To quantify this exposure, we define the following correlation function


(5.3) $$\begin{array}{lcr} & \xi (b,c)=\frac{\int_{0}^{L}\int_{0}^{L}b\;c\;dx\;dz}{\int_{0}^{L}\int_{0}^{L}bdxdz\int_{0}^{L}\int_{0}^{L}cdxdz}, & \end{array}$$

which, for convenience, does not include a factor of $L^{2}$ in the denominator. We can plot this over time for different values of non-dimensional diffusivity γ in order to see the effect of faster diffusion, and thus stronger temporal gradients, on the ability of a bacterial population to track a pulse. We see from figure 6 that the population achieves a fairly steady state at t=16, so we will restrict our simulations to a maximum non-dimensional time $\tau_{max}=15$. We will reintroduce concentration sensing thresholds, so that chemotaxis is limited at very high and very low concentrations.

**Figure 7.**
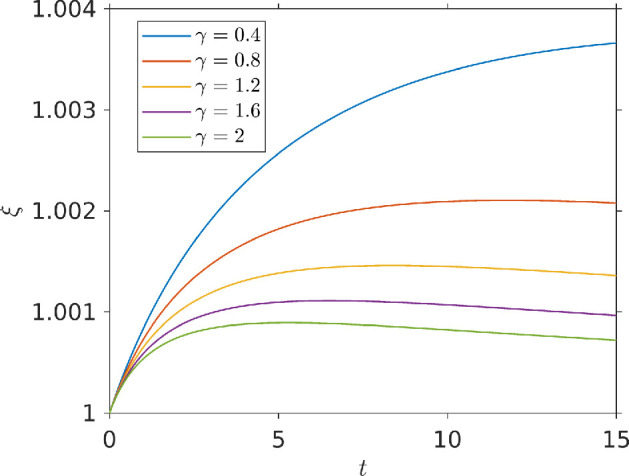
Chemo-effector exposure function plotted against time for a bacterial population responding to a pulse of chemo-attractant at the centre of a square domain. Results are shown for different values of the diffusivity parameter γ. Parameters used: $\tau_{max}=15$, $\zeta =0.5,\kappa_{c}=0.3104,V=1,\eta =5.7875,k=5,\sigma_{c}=2.5,v_{\infty }=0.63,c_{l}=0.1,c_{h}=15,L=100$.

Because varying chemo-effector diffusivities will alter temporal gradients, and with these exposure, we ran our simulation for different chemo-effector diffusivity strengths γ and then plotted our exposure function ξ(b,c) over time. Figure 7 shows how the exposure measure ξ changes with time for different values of the dimensionless chemo-effector diffusivity γ. At low diffusivities (γ=0.4) exposure grows monotonically over time. In this case, small γ means the pulse decays slowly: temporal gradients are weak, and bacteria are not very inhibited to chemotactically travel up the strong pulse gradient. For stronger diffusivities (γ≳0.8), exposure varies non-monotonically: we initially see a gain in chemo-effector exposure, but this then falls at longer times. This is because the pulse rapidly diffuses into the surrounding medium, creating strong temporal gradients, which inhibit chemotaxis up the pulse.

#### (i) Speed dependence of chemo-effector exposure

**Figure 8.**
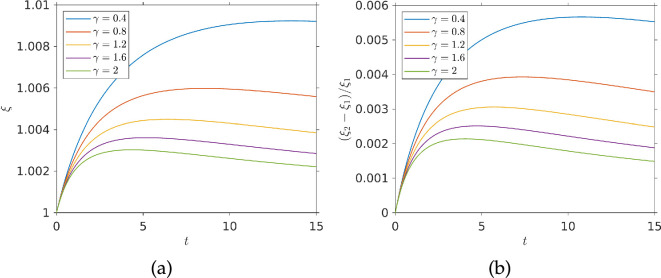
Effect of bacterial swimming speed on exposure: (*a*) exposure function from a swimming population with V=2 (twice the value in figure 7); (*b*) fractional difference in exposure, $(\xi_{2}-\xi_{1})/\xi_{1}$, between bacteria with swimming speed V=2 and V=1. All other parameters as in figure 7.

We have shown how temporal gradients can affect the accumulation of bacteria in response to pulses of chemoattractant, leading to diminished exposure at high diffusivities. Here we investigate varying swimming speed, which analyses of single bacteria have predicted can reduce the effect of temporal gradients on chemosensing [20]. To study the effect of swimming speed, we solved the chemo-effector pulse model with an increased non-dimensional swimming speed of V=2 to compare it with the previous simulation at half this speed (all other parameters kept the same). The result of this simulation can be seen in figure 8. Figure 8*a* shows the chemo-effector exposure over time with V=2 and figure 8*b* shows the fractional difference in exposure between a simulation with V=1 and one with V=2. As we can see, initially faster swimming bacterial populations rapidly develop an advantage over slower swimming bacteria at all diffusivities. The fractional difference in exposure grows slower and peaks sooner for higher diffusivities. At later times, when the pulse has decayed and chemo-effector concentration returns to equilibrium, the fractional difference in exposure begins to fall. This happens sooner for stronger diffusivities, when the pulse decays faster. In all cases, the fractional difference in exposure is very small, but this may not be the case over several pulses, as we shall next investigate.

### (b) Periodic chemo-effector pulses

To investigate the effect of repeated pulses, we modify our chemical transport equation given by (4.8) with a source term to give


(5.4) $$\frac{\partial c}{\partial t}=\gamma \nabla^{2}c+k|\sin(\omega t)|\exp\left(-\frac{[(x-L/2{)}^{2}+(z-L/2{)}^{2}{]}^{1/2}}{\sigma_{c}}\right),$$

where ω is the frequency of the pulses. Experimentally, this could be implemented by periodically releasing a chemo-effector into a reservoir via a pipette using a programmed pump. We chose uniform bacterial and chemo-effector concentrations for our initial conditions


(5.5) c(x,z,0)=1,b(x,z,0)=1.

**Figure 9.**
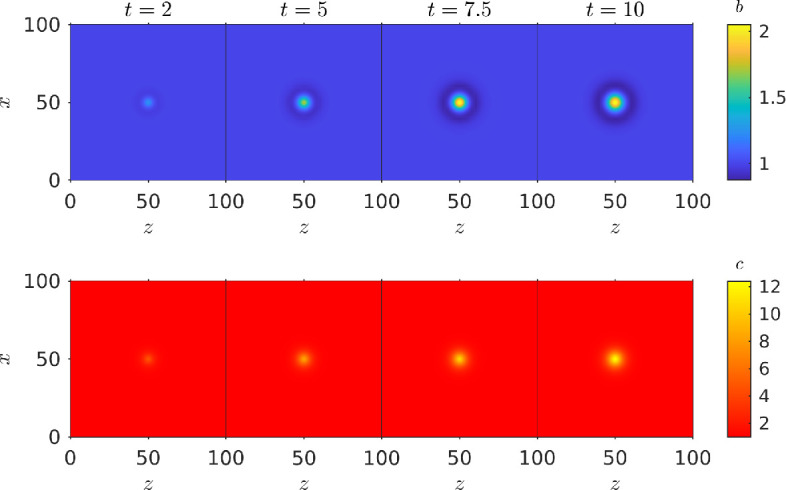
Bacterial population responding to periodic pulses of chemo-attractant in the centre of a square domain. Top row: bacterial concentration. Bottom row: chemo-effector concentration. Parameters used: $\tau_{max}=10$, $\zeta =0.5,\kappa_{c}=0.3104,V=1,\eta =5.7875,k=5,\sigma_{c}=2.5,v_{\infty }=0.63,c_{l}=0.1,c_{h}=15,\omega =\pi ,\gamma =0.8,L=100$.

Once again we ran our simulation for different chemo-effector diffusivity strengths γ, to evaluate the exposure function ξ(b,c) over time, and see how chemo-effector diffusion affects exposure. Snapshots of one of these simulations (γ=0.8) at selected time points are shown in figure 9. Bacteria are clearly seen to accumulate at the source of the chemical pulses in the centre of the domain.

**Figure 10.**
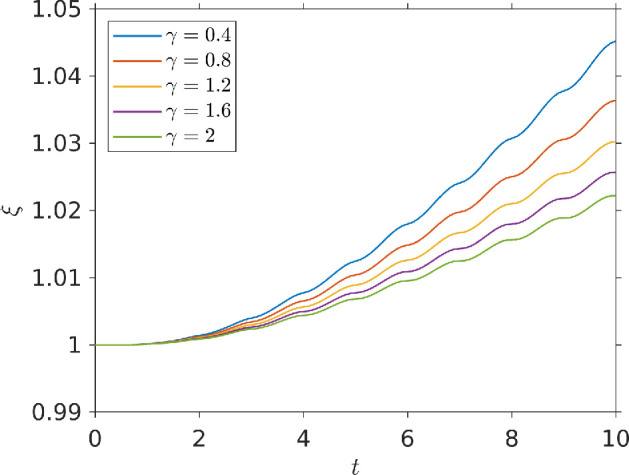
Chemo-effector exposure function plotted against time for a bacterial population in an environment where chemo-attractant is periodically pulsed at the centre of a square domain. Results are shown for different values of the diffusivity parameter γ. Parameters used: $\tau_{max}=10$, $\zeta =0.5,\kappa_{c}=0.3104,V=1,\eta =5.7875,k=5,\sigma_{c}=2.5,v_{\infty }=0.63,c_{l}=0.1,c_{h}=15,\omega =\pi ,L=100$.

Figure 10 shows how the exposure measure ξ changes with time for different values of the dimensionless chemo-effector diffusivity γ. We see that varying γ does not have much effect on nutrient exposure until t=1 (after the first pulse). Subsequently, exposure grows with time, rising faster for lower values of γ. Exposure grows larger after several pulses, as bacteria are able to accumulate at higher chemo-effector levels. The exposure curves display modulation, which reflects the effects of the periodic variation of the pulse. As this grows, positive temporal gradients enhance chemotaxis up the chemo-effector gradient, leading to strong bacterial accumulation. The chemical then builds up sufficiently that diffusion dominates its dynamics. Subsequently, as the pulse decays, temporal gradients inhibit chemotactic accumulation. For smaller diffusivities γ, the pulses do not dissipate so quickly. Bacteria still benefit from temporally enhanced chemotaxis as the pulse grows, but the temporal inhibition of chemotaxis due to chemo-effector diffusion in the pulse is diminished. This leads to greater accumulation and increased exposure. Weaker diffusivities also create stronger spatial gradients, which also enhance chemotactic accumulation and exposure.

#### (i) Speed dependence of chemo-effector exposure to a repeated pulse

**Figure 11.**
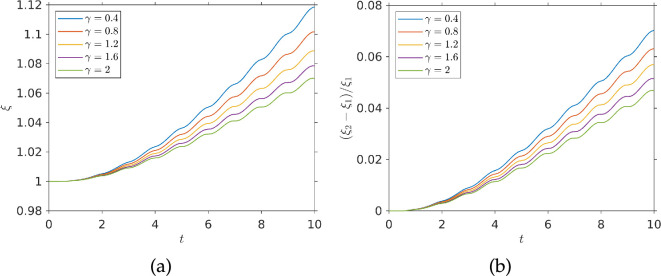
Effect of bacterial swimming speed on exposure: (*a*) exposure function from a swimming population with V=2 (twice the value in figure 10); (*b*) fractional difference in exposure, $(\xi_{2}-\xi_{1})/\xi_{1}$, between bacteria with swimming speed V=2 and V=1. All other parameters as in figure 10.

Following our approach to a single pulse, we re-ran the periodic chemo-effector pulse simulations with a non-dimensional swimming speed of V=2 to compare it with the previous simulation at half that speed (all other parameters kept the same). The result of this simulation can be seen in figure 11. Figure 11*a* shows the chemo-effector exposure over time and figure 11*b* shows the fractional difference in exposure between a simulation with V=1 and one with V=2. As we can see, for all diffusivities, the faster swimming population has a greater chemo-effector exposure and this difference grows over time. The fractional exposure difference shown in figure 11*b* displays some modulation.

This is due to the fact that faster bacteria are less susceptible to temporal gradients. When the pulse is strong enough to overpower diffusion, the slower bacteria are at an advantage as the positive temporal gradients are enhancing their chemotaxis more and *vice versa* when diffusion dominates creating negative temporal gradients, faster bacteria have their chemotaxis inhibited less and are at an advantage.

Therefore, we can say that in a dynamic chemo-effector environment with periodic chemo-effector pulses and our choice of parameters, faster swimming provides an advantage in seeking nutrients. We can see that this advantage is several orders of magnitude higher than the advantage accumulated during a singular pulse, as can be seen comparing figures 8*b* and 11*b*. Making the reasonable assumption that exposure correlates with nutrient uptake, we could then deduce that, for our choice of parameters, faster swimming bacteria could over time outcompete slower bacteria for resources. In the environment, source location would be subject to change and slower bacteria would likely be at an even larger disadvantage, as new pulses are sought.

## 6. Discussion

We have used Monte Carlo simulations to provide new predictions for the chemotactic drift velocity of a bacterial population in the presence of spatial and temporal chemo-effector gradients. We then constructed an ansatz for this drift velocity, which agrees well with the simulation data. Through further simulations we tested the dependence of the drift on swimming speed, as suggested by studies on individual bacteria [20], and found agreement between ansatz and simulation predictions. The expression for the drift velocity provided by our ansatz was then incorporated into a Patlak–Keller-Segel type continuum model and applied to two realistic examples of bacteria responding to temporally varying sources of chemo-effectors. In these cases, the exposure of bacteria to a chemo-effector, quantified by a correlation function, depends strongly on the source strength, but also on the diffusivity of the chemo-effector. In particular, strong temporal gradients can significantly reduce exposure.

Our simulation-backed theoretical framework provides the first population-level continuum model capable of describing bacteria in temporally varying landscapes, which are prevalent in natural and industrial environments. Laboratory-based experiments on chemotaxis have largely considered spatial chemical gradients that do not vary in time. It would be interesting to test our ansatz and our continuum model predictions using microfluidic experiments. For example, a pulsating source could be realized with a pipette tip connected to a pump periodically delivering a chemo-effector to a population of bacteria in a dilute agar gel.

There are a greater number of degrees of freedom involved in population-level chemotaxis in dynamic environments, as illustrated by our model. When only spatial gradients are present, one can have negative and positive chemotaxis up/down a gradient. However, as we have demonstrated, in the presence of temporal gradients, chemotaxis up a gradient can be enhanced or suppressed, depending on the gradient and bacterial parameters. Our discussion regarding exposure has been purposely general, without referring to long-term benefit or harm to bacterial populations. Clearly, however, if the chemo-effector is a nutrient, exposure could benefit growth. Conversely, if the chemo-effector is toxic, bacteria can be damaged and/or die. Our analysis implies that temporally varying gradients could reduce exposure to nutrients and enhance exposure to toxic chemicals, by an extent that depends on the diffusivity of these chemo-effectors. To explore this, it will be quite easy to amend our continuum model to include consumption/production of chemo-effector and growth/death of bacteria. This will allow the modelling of interesting scenarios in dynamic environments, such as competing bacterial populations with growth and death, or bacteria navigating concomitant gradients of chemo-effector and chemo-repellent.

Another interesting addition would be to include chemokinesis, a chemical-dependent change in swimming speed [25], into a Patak–Keller–Segel model using our new expression for the drift velocity. Our results suggest that faster swimming could offer a competitive advantage in dynamic environments. Many chemokinetic bacteria like *Vibrio* spp*.* live in marine environments where they are constantly navigating transient pulses of nutrients, such as those created by lysing algal cells [33]. It would be interesting to compare nutrient exposure for chemokinetic versus non-chemokinetic bacteria populations in those same environments. This could be simply implemented in our model by replacing the speed V with some chemo-effector-dependent speed function, since our model already allows for variable swimming speeds.

Many chemotactic bacteria display motility patterns different from *E. coli*’s run and tumble. For example, the marine bacterium *V. alginolyticus* ‘reverses and flicks’ [34]. It would thus also be interesting to recreate our Monte Carlo simulation based on these distinct motility patterns and chemotaxis, and observe how this influences our results for the chemotactic drift velocity.

## Data Availability

This manuscript has associated data in a data repository. Code and data associated with this manuscript are available from the Zenodo repository [35]. Supplementary material is available online [36].
